# Better mental health in children of Vietnamese refugees compared with their Norwegian peers - a matter of cultural difference?

**DOI:** 10.1186/1753-2000-3-34

**Published:** 2009-10-21

**Authors:** Aina Basilier Vaage, Laila Tingvold, Edvard Hauff, Thong Van Ta, Tore Wentzel-Larsen, Jocelyne Clench-Aas, Per Hove Thomsen

**Affiliations:** 1 Centre for Child and Adolescent Mental Health, University of Bergen, Bergen, Norway; 2 Department of Child and Adolescent Psychiatry, Stavanger University Hospital, Stavanger, Norway; 3 Institute of Psychiatry, University of Oslo, Oslo, Norway; 4 Oslo University Hospital, Ullevål Department of Psychiatry, Oslo, Norway; 5 International House Foundation, Stavanger, Norway; 6 Centre for Clinical Research, Haukeland University Hospital, Bergen, Norway; 7 Norwegian Institute of Public Health, Division of Mental Health, Oslo, Norway; 8 Centre for Child and Adolescent Psychiatry, University of Aarhus, Aarhus, Denmark

## Abstract

**Background:**

There are conflicting results on whether immigrant children are at a heightened risk of mental health problems compared with native youth in the resettlement country.

**The objective of the study:**

To compare the mental health of 94 Norwegian-born children from a community cohort of Vietnamese refugees, aged 4 - 18 years, with that of a Norwegian community sample.

**Methods:**

The SDQ was completed by two types of informants; the children's self-reports, and the parents' reports, for comparison with Norwegian data from the Health Profiles for Children and Youth in the Akershus study.

**Results:**

The self-perceived mental health of second-generation Vietnamese in Norway was better than that of their Norwegian compatriots, as assessed by the SDQ. In the Norwegian-Vietnamese group, both children and parents reported a higher level of functioning.

**Conclusion:**

This surprising finding may result from the lower prevalence of mental distress in Norwegian-Vietnamese children compared with their Norwegian peers, or from biased reports and cultural differences in reporting emotional and behavioural problems. These findings may represent the positive results of the children's bi-cultural competencies.

## Introduction

A frequently discussed question is whether immigrant children are at a heightened risk of mental health problems compared with native comparison groups. Reviews of the mental health of immigrant children and youth [1,2] have highlighted the conflicting results of different studies and the challenging nature of this field of research.

A factor that complicates any comparison is that the different groups of children included in these studies are often not adequately defined [1]. First, the definition of the group labelled "immigrant" covers a wide range of groups with very different histories concerning being uprooted from their home countries, migration and resettlement. Second-generation immigrants include both those children born in the country of origin but being very young at the time of their migration, and children born of migrant parents after arrival in the resettlement country . Consequently, "immigrant children" include some children who may have experienced a variety of adverse life events and been exposed to risk factors known to be related to the development of mental health problems.

Second, the lack of knowledge of the background of the immigrants' parents, such as where they came from and whether they were refugees, asylum-seekers or labour migrants, is a complication that may impact on their role as parents in a new country and thereby affect their children. Third, different informants and methods have been used in studies of children's emotional and behavioural problems. Cross-cultural differences in the perception of what constitutes mental health problems is an additional complicating factor [3].

Studies of the prevalence of mental disorders in children of Vietnamese refugees have reported contradictory results. Krupinski and Burrows [4] found a higher prevalence of mental disorders in children who recently immigrated to Australia compared with Australian-born children. Two years later, however, the prevalence was lower than in the general population. A more recent study of Vietnamese children and adolescents in Perth, Australia, [5] found the same prevalence of psychiatric disorders as in the general population.

A group of Vietnamese refugees, who arrived in Norway in 1982, were included in a prospective, longitudinal cohort study. The refugees first took part in the study on their arrival (T1), and they were followed up after three years (T2) and 23 years (T3). At T3 spouses and children born in Norway were also included in the study. The current study (T3, 2005-06) focuses on the mental health of these children, who were born in exile. This is the first European study focusing on the mental health of a group of second-generation immigrants, children of refugees, as reported by the children as well as by their parents.

The aim of the study was to compare the mental health of Norwegian-born children of Vietnamese refugees with that of a Norwegian community sample, using the Strengths and Difficulties Questionnaire (SDQ).

## Methods

### Design and procedures

The study reports cross-sectional data from a longitudinal, prospective cohort study of Vietnamese refugees arriving in Norway in 1982 [6].

A structured interview procedure was administered in the respondents' home by the first and fourth authors. The assessment of parents and children included structured self-report questionnaires and semi-structured interviews. Except for the SDQ, the questionnaires and the interviews were developed for this study. The children sat apart from their parents while they filled in the questionnaire and during the interview.

Written information about the study was provided in Vietnamese and Norwegian. The parents consented for their children to be included in the study, and both the parents and their children signed the consent form prior to the interviews. The study was approved by the Regional Committee for Medical Research Ethics and the Norwegian Social Science Data Services.

### Study populations

**Figure 1:**
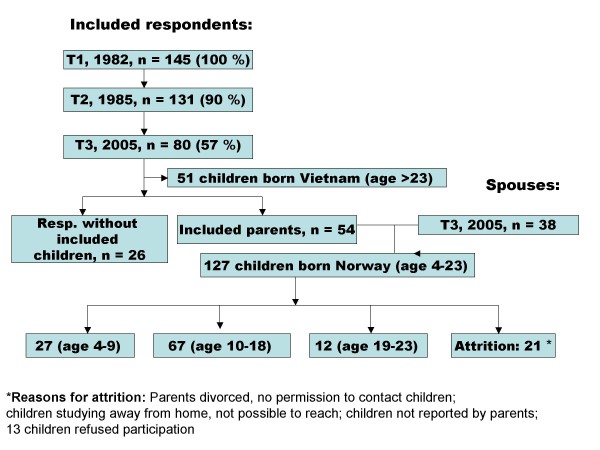
**Flow diagram of included Vietnamese refugees, spouses and children**.

*Children *(Figure 1. Flow diagram of included Vietnamese refugees, spouses and children)

The target population for this study was Norwegian-born children of Vietnamese refugees, here called the Norwegian-Vietnamese children.

Of the 103 children aged between 4 and 18 years who were eligible for inclusion in the study, we were able to include 94 (91%) children containing 51 girls and 43 boys (mean age: 11.8 years, SD: 3.9 years). Figure 1 shows the reasons for attrition of participants from the study. The younger group, 27 children aged between 4 and 9, were assessed indirectly, by means of parent assessment, while the other children were assessed directly using a self-report questionnaire and a semi-structured interview.

*Information from the parents *was available for 88 of the children included in the study, mainly from the mother.

*Information from T1 on the parents' *mental health was included for discussion of the "healthy migrant effect".

#### Population characteristics

All children lived with both parents, except for two single-mother families. The families lived in a geographically widespread area in the southern part of Norway, representing both urban and rural districts. The parents' main religious affiliation was Catholic (55%) or Buddhist (40%). The parents had 11.8 (SD 3.9) years of education. Permanent employment was reported by 67% of the parents. Ten per cent of the parents had temporary work and 10% were unemployed.

The parents spoke mainly Vietnamese with each other (ca 80%), while communication with the children was a combination of Vietnamese and Norwegian.

*One or both parents *belonged to the surviving cohort of refugees that was originally included in the study in 1982 and again in 1985 (see Figure 1). The refugees had been rescued by Norwegian merchant vessels from the South China Sea, and were given an offer to resettle in Norway. So, this original cohort may be regarded as a relatively unselected sample from the third wave of Vietnamese "boat people" [7], who fled the Vietnamese communist regime after the war in Vietnam. The parents of the children studied in the current report consisted of 38 mothers, whose mean age was 39.3 years (SD 5.5), and 45 fathers, whose mean age was 44.8 years (SD 4.8); all of these parents were Vietnamese, born in Vietnam. There were seven couples among the original respondents.

#### The Norwegian community comparison sample

Data from the Health Profiles for Children and Youth in the Akershus Study [8,9] were included in the study. The data were cross-sectional and based on self-reports and parent reports for a total of 36,465 children in Akershus county. Self-report data were available for 16,480 children in grades 3 to 7, and for 19,985 children in grades 8 to 13. Data were obtained from the parents of 14,698 children in grades 3 to 7. Mental health was assessed by the SDQ including the impact supplement, which provided self-report data for children in grades 5 to 13, and data from the parents of children in grades 3 to 7. Participation was anonymous and voluntary. The sub-sample of children (29,559, 85.6% of the total) who had two Norwegian-born parents served as a comparison group in the data analyses.

### Assessment of mental health

In the present study the children's mental health was assessed using the SDQ including the impact supplement [10,11]. The self-report questionnaire was used for all children aged between 10 and 18, in accordance with a Norwegian study [12], with parent reports for children aged from 4 to 18. The SDQ can be downloaded from .

We used the Norwegian cut-offs at the 80^th ^and 90^th ^percentiles from the Akershus study [12] for the SDQ total score and the subscale scores in order to categorize participants into a low-risk or normal group, a borderline group, or a high-risk or abnormal group. This categorization was used for both the Norwegian-Vietnamese children and the comparison group.

### The SDQ has been translated and used in a variety of cultures and language groups

We used the official Norwegian translation of the SDQ. As there was no official Vietnamese translation at the time of the study, in the Norwegian form for parents we included a Vietnamese translation in brackets to ensure understanding. The translation was performed in accordance with the cultural norms for translation [13].

The parents' mental health was assessed by the Symptom Check List Revised (SCL-90-R), as described elsewhere [6].

### Socio-demographic background for comparison of the two samples

For all included children we had information on the family situation, dichotomized to "living with both parents" and "other", and on the perceived level of family economy compared with other families in Norway (badly off/not so well, moderately well off or well/very well off). Parents' level of education and the families' yearly total income was reported for all the Vietnamese parents and for Norwegian parents of children grades 3-7.

### Statistical analysis

Gender and age group differences in the SDQ total score and subscales were tested by independent sample t-tests. An expected mean score [14] for each Norwegian-Vietnamese child was computed as the mean value in the Norwegian reference sample for children with the same gender and grade. Differences between the Norwegian-Vietnamese scores and the expected mean scores were tested by paired sample t-tests. As sensitivity analyses we repeated these tests with expected mean scores based also on family situation (whether the children were living together with both parents), and on perceived family economy, in addition to gender and grade.

The data from single items of the SDQ and the level of functioning distributions for the Norwegian-Vietnamese children were compared with data from the Norwegian reference sample using exact chi-square tests. Test results for single items are reported after Hochberg - Benjamini adjustment [15], because of the large number of items. To determine level of functioning, we used the Norwegian cut-off values. The exact chi-square tests used the Norwegian norm values as test values. The tests were adjusted for multiple testing by the Benjamini-Hochberg procedure [15]

The level of significance was set at .05. Statistical tendencies were reported when p < .10. All analyses used SPSS versions 15 or 17 (SPSS Inc, Chicago, IL, USA) and StatXact 8 (Cytel Inc., Cambridge, MA, USA).

## Results

### Comparison of socio-demographic background

The Norwegian-Vietnamese children were living together with both parents to a larger extent than the Norwegian children did (grades 3-7: 97.3% vs. 73.3%, grades 8-13: 97.2% vs. 67.4%). The Vietnamese parents had lower levels of education, their yearly total income was lower and the perceived level of family economy was more moderate than in the Norwegian families (Table 1).

**Table 1:** Socio-demographic background of the Norwegian (No) norm sample and the Norwegian-Vietnamese (NV) sample

**Parents' education**	**No, grades 3-7**		**NV, grades 3-7**		**NV, total, grades 3-13**	
	**% (n)**	**% (n)**	**% (n)**	**% (n)**	**% (n)**	**% (n)**
	**Mother** **n = 12,547**	**Father** **n = 12,343**	**Mother** **n = 28**	**Father** **n = 39**	**Mother** **n = 47**	**Father** **n = 68**
< 7 years	0	0	14.3 (4)	28.2 (11)	12.8 (6)	20.3 (14)
Primary school	2.7 (345)	2.9 (355)	21.4 (6)	12.8 (5)	25.5 (12)	10.3 (7)
Secondary school	9.4 (1180)	11.4 (1401)	17.9 (5)	33.3 (13)	17.0 (8)	30.9 (21)
Upper secondary (vgs^a^)	49.7 (6230)	45.6 (5625)	17.9 (5)	10.3 (4)	21.3 (10)	13.2 (9)
Tertiary education	38.2 (4792)	40.2 (4962)	28.6 (8)	15.4 (6)	23.4 (11)	25.0 (17)

**Family's yearly total income, NOK**	**No, grades 3-7** **% (n)**		**NV, grades 3-7** **% (n)**		**NV total, grades 3-13, % (n)**	

< 200.000	2.8 (350)		7.7 (3)		5.7 (4)	
200-400.000	21.6 (2659)		61.5 (24)		45.7 (32)	
400-600.000	36.8 (4539)		17.9 (7)		28.6 (20)	
> 600.000	38.8 (4787)		12.8 (5)		20.0 (14)	

**Perceived level of family economy**	**No, grades 3-7 %(n)**	**No, grades 8-13 %(n)**	**NV, grades 3-7 %(n)**	**NV, grades 8-13, %(n)**		

Badly off/not so well	10.7 (1339)	7.0 (1188)	10 (4)	3.2 (1)		
Moderately well off	46.6 (5845)	31.2 (5248)	85 (34)	71.0 (22)		
Well/very well off	42.7 (5354)	61.8 (10.399)	5 (2)	25.8 (8)		

^a ^Vgs, "Videregående skole" in Norwegian

### The SDQ results for Norwegian-Vietnamese children

We first analysed the SDQ total score, subscale scores and impact scores for all the Norwegian-Vietnamese children included in the study, as well as separately for girls and boys, based on the children's self -report data, and the parents' reports, as shown in Table 2.

**Table 2:** SDQ, self- and parent reports (total score, subscales and impact)^a^

	**All** **Mean (SD)**	**Girls** **Mean (SD)**	**Boys^b^** **Mean (SD)**
**Self-report SDQ**	n = 59	n = 28	n = 31
Total difficulties	9.3 (4.6)	9.4 (4.3)	9.1 (5.0)
Emotion	2.9 (2.2)	3.4 (2.2)	2.5 (2.1)
Conduct	1.6 (1.2)	1.3 (1.0)	1.9 (1.3)*
Hyperactivity	3.0 (2.1)	3.0 (2.1)	3.1 (2.2)
Peer problems	1.9 (1.5)	1.9 (1.5)	1.9 (1.6)
Prosocial	7.9 (1.7)	8.2 (1.5)	7.6 (1.9)
Impact	0.20 (0.66)	0.21 (0.79)	0.19 (0.54)
			
**Parent SDQ**	n = 88	n = 39	n = 49
Total difficulties	9.0 (5.7)	8.7 (5.7)	9.2 (5.8)
Emotion	2.5 (2.6)	2.7 (2.6)	2.3 (2.6)
Conduct	1.3 (1.3)	1.0 (1.1)	1.4 (1.4)
Hyperactivity	3.1 (2.1)	2.7 (2.2)	3.3 (2.0)
Peer problems	2.2 (1.5)	2.3 (1.5)	2.2 (1.4)
Prosocial	8.3 (1.6)	8.9 (1.1)	7.8 (1.9)**
Impact	1.82 (6.4)	2.18 (7.2)	0.86 (3.3)

^**a **^Higher scores indicate more problems on all scales except prosocial; high prosocial score indicate good function. An impact score of 1 is defined as borderline, 2 or more defined as abnormal or "caseness" according to Goodman [10].

^**b **^Significance for gender differences: * p-value < 0.05, ** p-value < 0.01

There were no significant differences when the age variable was dichotomized (children from preschool to grade 7 and adolescents from grade 8 to grade 13).

### Comparison of self-reports

Comparisons with a Norwegian control sample of children born of two Norwegian parents were possible for the self-reports of children in grades 5 to 13 (aged from 10 to 18 years) (Table 3). The Norwegian-Vietnamese comparison group included 53 of the 59 self-reports.

**Table 3:** SDQ total, subscales and impact, observed in Norwegian-Vietnamese children (NV), compared with expected mean scores from the Norwegian norm sample (No).

**SDQ**	**Total mean (SD)**		**Girls mean (SD)**		**Boys mean (SD)**	
**Self**	**NV (n = 53)**	**No**	**NV (n = 28)**	**No**	**NV (n = 25)**	**No**
Total problems	8.9 (4.4)*	10.5 (0.5)	9.4 (4.3)	10.6 (0.6)	8.3 (4.6)	10.4 (0.4)
Emotion	2.8 (2.2)	2.7 (0.6)	3.4 (2.2)	3.2 (0.2)	2.1 (2.0)	2.1 (0.1)
Conduct	1.5 (1.2)**	2.0 (0.3)	1.3 (1.0)	1.8 (0.1)	1.9 (1.2)	2.3 (0.2)
Hyperact	2.9 (2.1)***	4.0 (0.3)	3.0 (2.1)	4.0 (0.4)	2.8 (2.2)	4.0 (0.2)
Peer problems	1.8 (1.5)	1.9 (0.2)	1.9 (1.5)	1.7 (0.1)	1.8 (1.6)	2.0 (0.2)
Prosocial	7.9 (1.7)^t^	7.5 (0.6)	8.2 (1.5)	8.0 (0.2)	7.6 (1.8)^t^	6.9 (0.4)
Impact	0.2 (0.6) ***	0.9 (0.6)	0.2 (0.8)	1.0 (0.6)	0.1 (0.4)	0.7 (0.4)

**Parent**	**(n = 39)**		**(n = 23)**		**(n = 16)**	
Total problems	8.6 (6.2)*	6.3 (0.5)	7.8 (5.5)	5.7 (0.2)	9.1 (6.7)	6.7 (0.2)
Emotion	2.1 (2.5)^t^	1.3 (0.1)	1.9 (2.0)	1.4 (0.01)	2.2 (2.8)	1.2 (0.1)
Conduct	1.2 (1.4)	1.1 (0.1)	0.9 (1.1)	1.0 (0.02)	1.3 (1.5)	1.2 (0.05)
Hyperact	3.1 (2.4)	2.7 (0.4)	2.9 (2.7)	2.3 (0.2)	3.3 (2.2)	3.1 (0.1)
Peer problems	2.2 (1.5)***	1.1 (0.1)	2.1 (1.3)	1.1 (0.04)	2.2 (1.7)	1.2 (0.1)
Prosocial	8.3 (1.6)	8.2 (0.3)	9.1 (1.1)	8.6 (0.02)	7.8 (1.7)	8.0 (0.1)
Impact	1.1 (3.8)	0.4 (0.1)	0.5 (2.0)	0.3 (0.02)	1.4 (4.6)	0.5 (0.04)

Self-report grade 5-13, parent report grade 3-7.

^a ^Significance for difference from expected mean scores: ^t ^p-value 0.05-0.09; * p-value <0.05; ** p-value <0.01; *** p-value <0.001

Compared with their Norwegian peers, the scores obtained by the Norwegian-Vietnamese children were significantly lower on externalizing scales, including the impact scale. This general trend was unchanged in sensitivity analyses using expected mean scores based on additional characteristics (Table 4), and when the analyses were repeated separately for each gender.

**Table 4:** Analyses of SDQ total, subscales and impact observed in Norwegian-Vietnamese children (NV), compared with expected mean scores from the Norwegian norm sample (No), adjusted for age, gender, family and perceived level of economy.

**SDQ**	**Age and grade**		**Age, grade and family**		**Age, grade, family and perceived economy**	
**Self**	**NV (n = 53)**	**No**	**NV (n = 53)**	**No**	**NV (n = 49)**	**No**
Total problems	8.9 (4.4)*	10.5 (0.5)	8.9 (4.4)*	10.1 (0.5)	9.1 (4.5)**	10.9 (1.4)
Emotion	2.8 (2.2)	2.7 (0.6)	2.8 (2.2)	2.6 (0.6)	2.8 (2.2)	2.8 (0.7)
Conduct	1.5 (1.2)**	2.0 (0.3)	1.5 (1.2)*	1.9 (0.3)	1.6 (1.2)*	2.0 (0.3)
Hyperact	2.9 (2.1)***	4.0 (0.3)	2.9 (2.1)	3.3 (0.9)	2.9 (2.2)*	3.6 (1.1)
Peer problems	1.8 (1.5)	1.9 (0.2)	1.8 (1.5)	1.5 (0.4)	1.9 (1.5)	1.7 (0.6)
Prosocial	7.9 (1.7)^t^	7.5 (0.6)	7.9 (1.7)^t^	7.4 (0.6)	7.9 (1.7)*	7.3 (0.7)
Impact	0.2 (0.6) ***	0.9 (0.6)	0.2 (0.6)***	0.8 (0.5)	0.2 (0.7)***	0.9 (0.6)
**Parent**	**(n = 39)**		**(n = 39)**		**(n = 38)**	
Total problems	8.6 (6.2)*	6.3 (0.5)	8.6 (6.2)**	5.8 (0.7)	8.7 (6.3)*	6.3 (0.9)
Emotion	2.1 (2.5)^t^	1.3 (0.1)	2.1 (2.5)*	1.2 (0.1)	2.2 (2.5)*	1.3 (0.2)
Conduct	1.2 (1.4)	1.1 (0.1)	1.2 (1.4)	1.0 (0.1)	1.2 (1.4)	1.1 (0.2)
Hyperact	3.1 (2.4)	2.7 (0.4)	3.1 (2.4)	2.6 (0.5)	3.2 (2.4)	2.8 (0.6)
Peer problems	2.2 (1.5)***	1.1 (0.1)	2.2 (1.5)***	1.0 (0.1)	2.2 (1.5)***	1.1 (0.2)
Prosocial	8.3 (1.6)	8.2 (0.3)	8.3 (1.6)	8.2 (0.3)	8.3 (1.6)	8.1 (0.3)
Impact	1.1 (3.8)	0.4 (0.1)	1.1 (3.8)	0.3 (0.1)	1.1 (3.8)	0.3 (0.2)

Self-report grade 5-13, parent report grade 3-7. Total mean (SD).

^a ^Significance for difference from expected mean scores: ^t ^p-value 0.05-0.09; * p-value <0.05; ** p-value <0.01; *** p-value <0.001

Analyses of data from single items of the SDQ showed significant differences between the Vietnamese group and the Norwegian controls in one or two single items from all subscales; except for the self-report prosocial subscale. The main finding was that the Norwegian-Vietnamese children, to a large extent, reported views that were opposite to those of their parents, as they were less obedient (mean 0.63 vs. 1.41, reverse coding, p < .001), while the levels of happiness, restlessness and being "better with adults" were similar to those of their Norwegian peers. In addition, the Norwegian-Vietnamese children reported significantly more fears (mean 0.53. vs. 0.41, p = .04) and loneliness (mean 0.58 vs. 0.47, p = .04) than the Norwegian children.

### Comparison of parent reports

Comparisons with a Norwegian control sample were possible for the parents' reports of children in grades 3 to 7 (aged from 8 to 12 years). The Norwegian-Vietnamese comparison group included 39 of the 88 parent reports.

The parents rated their children higher than did parents in the Norwegian control group. As for self-reports, the general trend was unchanged in sensitivity analyses (Table 4) and when repeated separately for each gender.

Also for the parent reports, analyses of data from single items of the SDQ showed significant differences between the Vietnamese group and the Norwegian controls in one or two single items from all subscales. Vietnamese parents reported higher scores than did the Norwegian controls for all emotional and conduct items. There were significantly higher unhappiness scores (mean 0.49 vs. 0.23, p = .019) and almost significantly higher obedience scores (mean 1.77 vs. 1.52, reversed coding, p = .068). The Vietnamese parents reported significantly more restlessness (mean 0.59 vs. 0.35, p = .032) and being "better with adults" (mean 1.15 vs. 0.24, p < .001), as well as a greater prosocial willingness to offer volunteer help (mean 1.69 vs. 1.31, p = .005).

### Level of functioning, self- and parent reports

For all self- reports, there were more Norwegian-Vietnamese children in the low-risk group. The parents' reports had the same pattern, except for peer problems, where fewer Vietnamese parents scored their children in the normal group (79.5% vs. 84.6%). Adjusted for multiple testing, none of the differences were significant.

## Discussion

The main finding from this study was that the mental health of second-generation Vietnamese in Norway, assessed by the children themselves, is better than that of their Norwegian compatriots. Norwegian-Vietnamese children and their parents reported greater levels of low-risk or normal functioning, although the parents reported that their children had more total problems and problems with peers than did parents in a Norwegian comparison study.

Contradictory results from studies of the mental health of Vietnamese children in exile suggest that our study belongs to a research field with many controversies.

Studies of immigrant mental health have been criticized for their lack of information on the mental health of the inhabitants of the country of origin [1,16]. Analyses of the Achenbach Child Behavior Checklist (CBCL) data in a population-based survey of mental health problems in Vietnamese children in Hanoi [5] showed that the Vietnamese children had lower scores than the US norms for this test, with only half as many scoring in the clinical range. Their result is consistent with our findings.

The discovery of better mental health in our study may have three different interpretations.

**First**, the results may indicate a true difference between Norwegian-Vietnamese and Norwegian children, as the lower prevalence of mental problems in Norwegian-Vietnamese children concurs with the results of other studies of South-East Asian immigrant children who have been assessed by the CBCL [17,18] or by the Rutter Parent Questionnaire [4], a predecessor of the SDQ. The CBCL and the SDQ are both designed to obtain ratings of children's problems and can be used to identify high-risk children [16].

The distributions of SDQ scores are found to be similar across the Nordic countries [19], including Norway.

Beiser et.al. [20] report better mental health in children of immigrants; this is partly attributed to Canada's selection - policy, "helping to ensure selection of healthy, resilient ...families and children". A "healthy immigrant effect" has been described, e.g. in studies from Canada [21,22], which has a large contingent of immigrants and an immigration selection-policy. After arriving as apparently healthy immigrants [23], the health of immigrants subsequently declines and converges towards the native- born population. Contrary to this, the unselected Vietnamese parents of the study sample arrived in Norway with higher levels of psychological distress than in the host-populations [6], 1/4 scoring as "cases". Norway had no pre-existing South East Asian cultural community and none of the refugees had any knowledge of the Norwegian language prior to their escape from Vietnam. As a group, they were relatively unprepared for migration, and the changes represented large-scale acculturative stress. Consequently, the finding of good mental health in the refugees' children in our study cannot be explained by the "healthy immigrant effect".

Some aspects of the Vietnamese children's family life may account for a lower prevalence of mental illness. Possible protective factors include a family structure firmly rooted in a tradition and value system [17,24], as well as parental supervision [25]. Cross-cultural differences in socialization practices and expectations for children's behaviour [5,26] may cause Vietnamese parents to discourage externalizing behaviours more forcefully in their children. Even so, the children in our study had levels of self-rated emotional problems comparable to their Norwegian counterparts. Thus, our findings may indicate an immigrant advantage in terms of emotional and well-being outcomes.

Other factors that should be considered include genetic factors, temperamental differences [18] and the parents' health [27]. The relationships between the parents' and the children's health will be reported in a forthcoming paper.

**Second**, the reports of good mental health may be biased. As a consequence of the high expectations concerning their behaviour and performances, and the upbringing in a culture in which mental illness is highly stigmatized [28], immigrant adolescents may feel less comfortable reporting behaviours that might be perceived as deviant. Such social desirability may be seen as a bias, as well as an adaptation to Vietnamese cultural and parental values.

Surprisingly, we found that the parents reported as much disruptive behaviour as the Norwegian community sample, and some scores were even higher, especially the number of peer problems (Table 2). Being less acculturated than their children, immigrant parents may be mostly at a loss when evaluating peer relationships in the Norwegian cultural context. Parents worry that their children are not working hard enough to achieve academic success [29]. This may explain the parents' reports of high levels of problems in their children as possible instances of over-reporting.

**Third**, the Norwegian-Vietnamese children, but especially their parents, may understand the statements in the SDQ differently from Norwegians, parallel to the conclusion in a Chinese study [30]. Assumptions about development, normality and psychopathology are culturally embedded [31,32], and there are cultural differences in definitions of psychopathology [33]. In his studies, McKelvey [3,34] mentions that, despite the CBCL's impressive performance in several cross-cultural settings [35], symptoms that are possibly related to child mental illness may have a different meaning within the Vietnamese cultural context. The higher parent-rated problem scores in our study may reflect the parents' critical or anxious monitoring of their children's school performances, more so than reflecting any symptoms of psychopathology.

Stevens et al. [1] discussed the validity of cross-cultural assessment. Although several studies indicated that their instrument showed sufficient validity for their populations, as comparable factor structures and high reliabilities for both the migrant and the native populations were revealed [36], the instruments used may be less valid for assessing migrant samples. This explanation of the differences in problem behaviour between migrant and native youth has been supported by others utilizing the SDQ [37,38].

### Strengths and limitations

This research formed part of a prospective longitudinal follow-up study. The personal follow-up design of the study was strengthened by a culturally relevant approach enacted by the Vietnamese co-researcher. As he was responsible for making contact with the families, his efforts contributed to the high inclusion rate of children (91%), which is considered a major strength of the study. The longitudinal prospective design, with information on the parents' mental health, is another strength.

Additional strength is the use of two informants. The discussion on what type of informant carries the highest weight is ongoing [39]. Montgomery [40] wondered whether the Youth Self Report (YSR) and the CBCL might be considered as measuring two qualitatively different constructs, with the difference between informants not just resulting from cross-informant disagreement. This difference is found to a higher degree in refugee- and immigrant populations [40], as in our study (to be reported elsewhere). A similar question may be posed for the reports from the SDQ, as from the CBCL/YSR. As a group, children of Vietnamese refugees are higher acculturated than their parents [41]. Consequently, comparison of self-reports may be considered as more culturally relevant than a comparison of parents' reports for the two samples.

One important limitation of the study is its small sample size that requires a cautious interpretation of the findings. It made it difficult to adjust for the number of children in some families, as siblings' reports cannot be considered as independent. However, the small sample is from an unselected group of refugee parents. Countries with a large immigrant population, as Canada, have immigrant selection policies probably resulting in a different composition of immigrants, also in terms of mental health. The results from a non-selected group of refugee-families, although small, can therefore also be considered as strength of the study.

A major advantage as well as a challenge of the study was the comparison of the Norwegian-Vietnamese and the Norwegian community samples. Some basic information available in both samples on the families, including the parents' income and perceived economy, made possible a sensitivity analysis of the comparison of two samples, using somewhat broader information than just gender and grade. On one hand, the Norwegian - Vietnamese children were to a higher degree than their peers living together with both parents, a fact expected to explain a better mental health in the children [42]. On the other hand, the lower level of education as well as economy in the Vietnamese families would expectedly result in worse mental health in the children [43]. Still, basing our comparison on all these variables, the pattern of better mental health in the Norwegian-Vietnamese sample persisted.

A limitation of the study is the lack of comparison groups for the whole age range included in the study, that is, for both the self - reports and the parent reports.

A possible a limitation of the study is that the question whether the differences in mental health in the two samples can be explained by the cultural differences is still unanswered. The three different aspects described in the discussion-section are all, to some extent, related to the issue of "culture", and the role of migration and culture are difficult to disentangle from each other.

The lack of cultural validation of the assessment tools is a general problem that is not limited to this study and represents a major challenge in trans-cultural research.

The refugees studied at T3 were considered to be a representative sample of the third wave of boat refugees who arrived in Norway in 1982. The major characteristics of the parents included in the study were the same as those in the group who did not have children born in Norway. Consequently, and in spite of the reported limitations of the study, the children may be considered a representative sample of second-generation Vietnamese in Norway, who belonged to this group of Vietnamese refugees.

## Conclusion

The finding of lower self-rated mental health problem scores in Vietnamese-Norwegian children and their higher level of functioning when compared with a comparison group of Norwegian children was a surprise. The finding may result from the lower prevalence of mental distress in Norwegian-Vietnamese children or from biased reports and cultural differences in reporting emotional and behavioural problems.

The results may illustrate some positive aspects of the children's resilience and bicultural competencies, because migration might have a positive effect on Vietnamese children born in Norway. Studies of other aspects of the children's lives will be of importance when assessing some of the questions raised in this paper. How parents and children communicate about health and acculturation should be further explored by using qualitative methods.

## Competing interests

The authors declare that they have no competing interests.

## Authors' contributions

EH performed the two first studies of the Vietnamese refugees (1982 and 1985), planned the current study, and discussed the results and the draft. ABV and TVT planned the study, carried out the interviews and discussed the results. TWL and ABV conducted the statistical analyses. ABV prepared the manuscript. JCA was responsible for data from the Norwegian norm sample and discussion of the results. LT discussed the results. PHT planned the study and participated in the discussion of the results and the draft. All authors read and approved the final manuscript.

## References

[B1] Stevens GWJM, Vollebergh WAM (2008). Mental health in migrant children. Journal of Child Psychology & Psychiatry & Allied Disciplines.

[B2] Lustig SL, Kia-Keating M, Knight WG, Geltman P, Ellis H, Kinzie JD, Keane T, Saxe GN (2004). Review of child and adolescent refugee mental health. Journal of the American Academy of Child & Adolescent Psychiatry.

[B3] McKelvey RS, Baldassar LV, Sang DL, Roberts L (1999). Vietnamese parental perceptions of child and adolescent mental illness. Journal of the American Academy of Child & Adolescent Psychiatry.

[B4] Krupinski J, Burrows G (1986). The price of freedom Young Indochinese refugees in Australia.

[B5] McKelvey RS, Sang DL, Baldassar L, Davies L, Roberts L, Cutler N (2002). The prevalence of psychiatric disorders among Vietnamese children and adolescents. Medical Journal of Australia.

[B6] Hauff E, Vaglum P (1993). Vietnamese boat refugees: the influence of war and flight traumatization on mental health on arrival in the country of resettlement. A community cohort study of Vietnamese refugees in Norway. Acta Psychiatrica Scandinavica.

[B7] Hauff E (1998). The stresses of war, organised violence and exile: A prospective community cohort study of the mental health of Vietnamese refugees in Norway.

[B8] Rødje K, Clench-Aas J, van Roy B, Holmboe O, Muller A (2004). Helseprofil for barn og ungdom i Akershus - Ungdomsrapport.

[B9] Rødje K, Clench-Aas J, van Roy B, Holmboe O, Muller A (2004). Helseprofil for barn og ungdom i Akershus - Barnerapport.

[B10] Goodman R (1999). The extended version of the Strengths and Difficulties Questionnaire as a guide to child psychiatric caseness and consequent burden. Journal of Child Psychology & Psychiatry & Allied Disciplines.

[B11] Goodman R, Ford T, Simmons H, Gatward R, Meltzer H (2000). Using the Strengths and Difficulties Questionnaire (SDQ) to screen for child psychiatric disorders in a community sample. British Journal of Psychiatry.

[B12] Van Roy B, Groholt B, Heyerdahl S, Clench-Aas J (2006). Self-reported strengths and difficulties in a large Norwegian population 10-19 years: age and gender specific results of the extended SDQ-questionnaire. European Child & Adolescent Psychiatry.

[B13] Matias-Carrelo LE, Chavez LM, Negron G, Canino G, Aguilar-Gaxiola S, Hoppe S (2003). The Spanish translation and cultural adaptation of five mental health outcome measures. Culture, Medicine & Psychiatry.

[B14] Fayers P, Machin D (2007). Clinical interpretation. Population norms. Quality of life The assessment, analysis and interpretation of patient-reported outcomes.

[B15] Benjamini Y, Hochberg Y (1995). Controlling the false discovery rate: A practical and powerful approach to multiple testing. Journal of the Royal Statistical Society Series B.

[B16] Achenbach TM, Becker A, Dopfner M, Heiervang E, Roessner V, Steinhausen H-C, Rothenberger A (2008). Multicultural assessment of child and adolescent psychopathology with ASEBA and SDQ instruments: research findings, applications, and future directions. Journal of Child Psychology & Psychiatry & Allied Disciplines.

[B17] Rousseau C (2000). Living conditions and emotional profiles of Cambodian, Central American, and Quebecois youth. Canadian Journal of Psychiatry.

[B18] Chang L, Morrissey RF, Koplewicz HS (1995). Prevalence of psychiatric symptoms and their relation to adjustment among Chinese-American youth. Journal of the American Academy of Child & Adolescent Psychiatry.

[B19] Obel C, Heiervang E, Rodriguez A, Heyerdahl S, Smedje H, Sourander A, Guethmundsson OO, Clench-Aas J, Christensen E, Heian F (2004). The Strengths and Difficulties Questionnaire in the Nordic countries. European Child & Adolescent Psychiatry.

[B20] Beiser M, Hou F, Hyman I, Tousignant M (2002). Poverty, family process, and the mental health of immigrant children in Canada. American Journal of Public Health.

[B21] Ali JS, McDermott S, Gravel RG (2004). Recent research on immigrant health from statistics Canada's population surveys. Canadian Journal of Public Health Revue Canadienne de Sante Publique.

[B22] Newbold KB (2005). Self-rated health within the Canadian immigrant population: risk and the healthy immigrant effect. Social Science & Medicine.

[B23] McDonald JT, Kennedy S (2004). Insights into the 'healthy immigrant effect': health status and health service use of immigrants to Canada. Social Science & Medicine.

[B24] Huckans MS (2004). Family rituals, acculturation stress, and adjustment: A study of second-generation, adolescent, children of immigrants from Central America.

[B25] Harker K (2001). Immigrant generation, assimilation, and adolescent psychogical well-being. Social Forces.

[B26] Woo BSC, Ng TP, Fung DSS, Chan YH, Lee YP, Koh JBK, Cai Y (2007). Emotional and behavioural problems in Singaporean children based on parent, teacher and child reports. Singapore Medical Journal.

[B27] Youngstrom E, Loeber R, Stouthamer-Loeber M (2000). Patterns and correlates of agreement between parent, teacher, and male adolescent ratings of externalizing and internalizing problems. Journal of Consulting & Clinical Psychology.

[B28] Lee E (1988). Cultural factors in working with Southeast Asian refugee adolescents. Journal of Adolescence.

[B29] Boehnlein JK, Tran HD, Riley C, Vu KC, Tan S, Leung PK (1995). A comparative study of family functioning among Vietnamese and Cambodian refugees. Journal of Nervous & Mental Disease.

[B30] Du Y, Kou J, Coghill D (2008). The validity, reliability and normative scores of the parent, teacher and self report versions fo the Strengths and Difficulties Questionnaire in China. Child and Adolescent Psychiatry and Mental Health.

[B31] DiNicola V (1998). Children and families in cultural transition.

[B32] Kinzie JD (2001). Cross-cultural treatment of PTSD.

[B33] Davies LC, McKelvey RS (1998). Emotional and behavioural problems and competencies among immigrant and non-immigrant adolescents. Australian & New Zealand Journal of Psychiatry.

[B34] McKelvey RS, Davies LC, Sang DL, Pickering KR, Tu HC (1999). Problems and competencies reported by parents of Vietnamese children in Hanoi. Journal of the American Academy of Child & Adolescent Psychiatry.

[B35] Crijnen AA, Achenbach TM, Verhulst FC (1997). Comparisons of problems reported by parents of children in 12 cultures: total problems, externalizing, and internalizing. Journal of the American Academy of Child & Adolescent Psychiatry.

[B36] Wissink IB, Dekovic M, Meijer AM (2006). Parenting behavior, quality of the parent-adolescent relationship, and adolescent functioning in four ethnic groups. Journal of Early Adoilescence.

[B37] Leavey G, Hollins K, King M, Barnes J, Papadopoulos C, Grayson K (2004). Psychological disorder amongst refugee and migrant schoolchildren in London. Social Psychiatry and Psychiatric Epidemiology.

[B38] Oppedal B, Røysamb E, Heyerdahl S (2005). Ethnic group, acculturation, and psychiatric problems in young immigrants. Journal of Child Psychology and Psychiatry.

[B39] Becker A, Hagenberg N, Roessner V, Woerner W, Rothenberger A (2004). Evaluation of the self-reported SDQ in a clinical setting: Do self-reports tell us more than ratings by adult informants?. European Child & Adolescent Psychiatry.

[B40] Montgomery E (2008). Self- and parent assessment of mental health: disagreement on externalizing and internalizing behaviour in young refugees from the Middle East. Clinical Child Psychology & Psychiatry.

[B41] Chun KM, Akutsu PD, Trinh N-H, Rho YC, Lu FG, Sanders KM (2009). Assessing Asian American family acculturation in clinical settings: Guidelines and recommendations for mental health professionals. Handbook of mental health and acculturation in Asian American families.

[B42] Storksen I, Roysamb E, Holmen TL, Tambs K (2006). Adolescent adjustment and well-being: effects of parental divorce and distress. Scandinavian Journal of Psychology.

[B43] Costello EJ, Compton SN, Keeler G, Angold A (2003). Relationships between poverty and psychopathology: a natural experiment [see comment]. JAMA.

